# Study on R&D result subsidy strategies for PEV enterprises based on heterogeneous consumer technology thresholds and preferences under anxiety issues

**DOI:** 10.1371/journal.pone.0314476

**Published:** 2025-02-18

**Authors:** Ye Wang

**Affiliations:** School of Government, Henan University of Economics and Law, Zhengzhou, China; Institution of Eminence (IoE), University of Delhi, INDIA

## Abstract

Many governments worldwide hoped to stimulate Pure Electric Vehicle(PEV) enterprises’ R&D and sales through R&D result subsidy policies, but the anxiety issues significantly reduced PEV sales and weakened the policy effectiveness. To achieve better incentive effects, considering the impact of anxiety issues on subsidy strategies is necessary. As anxiety problems stem from typical behavior characteristics of PEV consumers——consumer technology thresholds, reasonable study should understand them and quantify their impact from the perspective of consumer technology thresholds. Therefore, it constructs a sequential game model among the government, the PEV company, and consumers with two dimensions of heterogeneous behavior characteristics——technology thresholds and preferences. It also determines the optimal R&D result subsidy strategies and analyzes the impact of the technology thresholds and preferences on them. It shows that the government should provide subsidies except for PEV enterprises with R&D efficiency in the higher range, and its optimal strategies must consider consumer technology threshold and preference conditions. The lower the technology thresholds of PEV consumers, the lower the optimal subsidy ratio until the technology level of the enterprise is already high enough, and there is no need for subsidies. Higher consumer technology preferences of PEV consumers will achieve the same effect. The numerical simulation shows that compared to other models, the model considering PEV consumer technology thresholds can optimize the subsidy ratio and achieve better incentive effects.

## Introduction

Many governments worldwide hoped their R&D result subsidy policies would spur R&D and sales of PEV (Pure Electric Vehicle) enterprises in their countries. However, the anxiety problems have significantly reduced sales and weakened the policy’s effectiveness [1–3]. For example, the Chinese government has subsidized its PEV enterprises to incentivize their R&D and sales since 2010, but the policy did not achieve the expected effect until 2017 [4, 5]. After the last eight years of subsidies, the technical level of the PEV range was still low, and the PEV market share was less than 3% in China [6, 7]. In contrast, anxiety issues, such as range anxiety, were always the most concerning issues for PEV consumers, which had held back the technological innovation and spread of PEVs [5, 8]. Therefore, it is essential to consider anxiety when a government implements an R&D result subsidy policy for PEV enterprises [9, 10].

Although anxiety issues are significant in the PEV market, few studies pay attention to them among influence factors of effects of a government R&D subsidy policy, and more studies focus on influence factors such as enterprise, subsidy, and industry characteristics (Table 1).

**Table 1:** Research on influence factors of government R&D subsidy effect.

Influence factors	Types	Authors and Publication Year
Enterprise types	Enterprise characteristics	Steinmo et al. [11], 2022
Technology capacity		Zhang and Huang [12], 2021
R&D patterns		Shao and Hua [13], 2023
Financial constraints		Li et al. [14], 2021
Risk attitudes		Huang et al. [15], 2024
Social responsibility		Liu et al. [16], 2021
Innovation subsidy vs. product subsidy	Subsidy characteristics	Li et al. [17], 2020
Fixed subsidy vs. discount subsidy		Zhang et al. [18], 2021b
R&D subsidy vs. non-R&D subsidy		Zhao [19], 2024
Reduction way		Ye et al. [20], 2021
Withdrawal probability		Nagy et al. [21], 2021
Industry types	Industry characteristics	Shen and Lin [22], 2020
Concentration		Gao et al. [23], 2021

Meanwhile, a few studies on government subsidy strategies considered anxiety issues in the PEV market. These studies typically construe and solve a game theory model to obtain the optimal government subsidy strategies and consider the impact of anxiety issues in the consumer demand model section. For example, Li and Wang obtained the optimal electric vehicle subsidy decisions through a game model among a vehicle manufacturer, a social planner, and vehicle consumers and abstracted range anxiety as a coefficient that affects consumers’ evaluation of electric vehicles [3]; Xu, Li, and Li constructed a game model among a battery supplier, an electric vehicle manufacturer, a government and consumers, which evaluated consumers’ range anxiety by a demand sensitivity coefficient [24]. Abstracting the impact of consumer behavior characteristics as an adjustment coefficient of the demand model section in a game model is a standard method, such as the coefficients of consumer preference, consumers’ environmental awareness, and social responsibility [12, 25, 26]. However, this abstraction is not enough to reflect the essence of anxious issues, and a more essential understanding of anxiety issues is necessary [27–30].

A particular type of anxiety issue is not a behavior characteristic of PEV consumers but a reflection of consumer behavior characteristics. For example, range anxiety reflects the behavior characteristics of consumers with technology thresholds for the PEV range [31–34]. In other words, a PEV consumer will experience anxiety when the technology level of a PEV does not meet the minimum requirement, that is, the consumer technology threshold [35, 36]. Some studies have explored the existence and connotation of technology thresholds of PEV consumers. Hackbarth and Madlener found that the most crucial characteristic of alternative fuel vehicle consumers is that the vehicle features must meet some minimum requirements in Germany [32]. Eggers and Eggers found that most PEV consumers consider the limited range unacceptable [31]. Xie et al. found that a driver develops range anxiety when the actual range exceeds his perceived “comfortable” range [34, 37]. Noel et al. revealed why Nordic consumers have a high range threshold: Norwegians will require the range of a PEV to meet the needs of a round-trip trip when they want to go to a mountain cabin on vacation [38]. Therefore, only from the perspective of PEV consumers’ technology thresholds can studies fundamentally analyze anxiety issues and explore their impact.

Furthermore, it is uncommon to consider two dimensions of heterogeneous characteristics of consumers in the demand model section of a game model, which can make solving and explaining models complex. For instance, Tang and Liang focused on identical consumers’ environmental awareness, while Hong et al. were concerned with the same green preference in production decisions [25, 26]. Zhang and Huang assumed the heterogeneous value appraisal and identical consumer social responsibility in vehicle product-line strategies [12]. Li and Wang are concerned about the willingness to pay, the anxiety degree, the environmental preference, and the green attribute of PEV consumers in PEV price and production decisions; although both the willingness to pay and the green attribute are heterogeneous, the latter only distinguish between green and non-green consumers [3].

This paper presents four key contributions. Firstly, it attempted to explain the anxiety problems from the perspective of PEV consumer behavior characteristics, that is, PEV consumer technology thresholds, and constructed the demand model section of the game model, which is an improvement on the demand model of PEV consumers. Secondly, it constructed the demand model of PEV consumers by considering two dimensions of heterogeneous behavior characteristics, which better describe PEV consumers and is another improvement to the demand model of PEV consumers. Thirdly, it obtained the optimal R&D result subsidy strategies considering the impact of PEV consumer technology thresholds and preferences, that is, the influence of anxiety issues, whose conclusions are more reliable as it has considered anxiety issues more fundamentally. Fourthly, it answered the impact of anxiety issues on the R&D subsidy policy from the perspective of PEV consumer technology thresholds, which supplements and expands the research on influence factors of the government R&D subsidy policy.

This study consists of seven sections. Section 2 provides the problem description, the hypothesis, and the model. Section 3 presents the solutions of the optimal subsidy strategies. Section 4 analyzes the influence of consumer technology thresholds and technology preferences on subsidy strategies. Section 5 conducts the numerical simulation. Sections 6 and 7 discuss and conclude.

## The model

### Problem description

PEV consumers have different technology thresholds and preferences as their typical behavior characteristics in the PEV market. The technology threshold is the minimum requirement of a consumer for a particular PEV technology or performance. If the technology or performance of the PEV does not reach the consumer technology threshold, the consumer will not buy it as useless. In other words, only if the technology of the PEV meets the consumer technology threshold may the consumer consider purchasing. For example, PEV consumers require that the range on a single charge is not less than their travel distance from the United States, the United Kingdom, and the 5 Nordic countries [38, 39]. Meanwhile, consumer technology preference is the degree to which consumers favor a PEV technology or performance; in simple terms, the utilities per unit of PEV technology [40–42].

Each PEV consumer has a goal of purchasing a valuable and economical PEV, and if a PEV meets the requirements, the consumer will buy one; otherwise, the consumer will not buy any. The purchase is a two-stage decision based on the unique technology threshold and preference. In the first stage, the consumer judges whether a specific technology or performance of the PEV can achieve the technology threshold. If it reaches, the consumer thinks that the PEV is helpful and decides to go to the second stage of the decision; otherwise, the consumer gives up the purchase. In the second stage, the consumer compares the value and the price of the PEV. The consumer measures the PEV value by the technology preference, and if the value is not less than the price, the consumer will buy the PEV; otherwise, abandon it.

A PEV enterprise develops, produces, and sells PEVs in the market and sequentially makes R&D, production, and pricing decisions to maximize profits. The enterprise needs to consider conditions such as R&D efficiency and production costs. It also needs to consider consumer behaviors, such as consumer technology thresholds and preferences, and government behaviors, such as an R&D subsidy policy.

The government provides R&D result subsidies to PEV enterprises to incentivize their R&D and sales. Its subsidy method is ex-post; according to the technical level of the PEV developed by the enterprise, the government will give the enterprise a certain subsidy ratio after sales.

**Fig 1:**
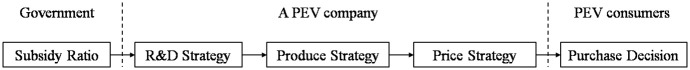
Content and sequence of decisions among the government, a PEV company, and PEV consumers.

Then, the government, the PEV enterprise, and consumers engage in a sequential game to achieve their respective goals in the PEV market. In the first stage, the government makes an R&D result subsidy decision, including whether to subsidize and the subsidy ratio. In the second stage, the PEV enterprise sequentially makes its R&D, production, and pricing decisions. In the last stage, consumers decide whether to buy a PEV. In general, the content and sequence of decisions are as follows (Fig 1):

### The model

#### Consumer purchase decision

Each PEV consumer has a unique technology threshold *t* and preference *θ* and decides whether to purchase a PEV based on them (see Table 2 for the summary of symbols and descriptions) [1, 43].

**Table 2:** The symbols and descriptions.

Symbols	Descriptions
*t*	Consumer technology threshold, *t* ~ *U*[0, *ϕ*]
*ϕ*	The highest technology threshold, *ϕ* ≥ 1
*θ*	Consumer technology preference, *θ* ~ *U*[0, *φ*]
*φ*	The highest technology preference, *φ* ≥ 1
*U*	Utility of a PEV consumer
*t*	The technology level of a PEV
*p*	Price of a PEV
*I*	R&D investment of an enterprise
*k*	R&D efficiency of an enterprise
$\bar{t}$	The ceiling of the technology level, $\bar{t}\leq \phi$
*c*	Production cost of a PEV
*s*	Ratio of R&D result subsidy
*π*	Profit of an enterprise

Assume the PEV developed by the enterprise has a technology level *t* and a price *p*. In the first stage of the decision, a consumer judges whether *t* ≥ *t* stands, which means the PEV is useful. If *t* ≥ *t*, the consumer further evaluates the economy of the PEV in the second stage; otherwise, the consumer gives up the purchase. In the second stage, the consumer judges whether *θt* ≥ *p* stands. If *θt* ≥ *p*, the consumer will buy a PEV; otherwise, the consumer will give up the purchase.

Then, the consumer purchase decision function as follows:

$$\left\{\begin{array}{ll} \begin{array}{ll} 1 & \underline{t}\leq t\;and\;U\left(t\right)\geq 0\\ 0 & or\;else \end{array} & and\;U\left(t\right)=\theta t-p \end{array}\right..$$ (1)

#### PEV demands

Assume the PEV market size is 1 for simplicity [7, 44]. The technology thresholds *t* of consumers are distributed uniformly from 0 to *ϕ* (*ϕ* ≥ 1), which means that the density of consumers on the technology threshold is 1/*ϕ*, and technology preferences *θ* are distributed uniformly from 0 to *φ* (*φ* ≥ 1), which means that the density of consumers on the technology preference is 1/*φ* [3, 10, 34, 37].

Assume the ceiling of the technology level $\bar{t}$ limits the technology level *t* due to the current basic science and technology level that is, $t\leq \bar{t}$. For all potential PEV consumers, only if their technology thresholds satisfy *t* ∈ [0, *t*] will they accept the PEV with technology level *t*. As the density of consumer technology thresholds is 1/*ϕ*, the proportion of consumers whose technology thresholds has been reached is $\int_{0}^{t}\frac{1}{\phi }d\underline{t}$. For these potential PEV consumers (*t* ∈ [0, *t*]), only if the PEV can bring non-negative utility, that is, *U*(*t*) = *θt* − *p* ≥ 0, the consumers will buy it. In other words, the above consumers with technology preferences *θ* ∈ [*p*/*t*, *φ*] will buy the PEV. As the density of consumer technology preferences is 1/*φ*, the proportion of consumers whose technology preferences have been reached is $\int_{\frac{p}{t}}^{\varphi }\frac{1}{\varphi }d\theta$.

**Fig 2:**
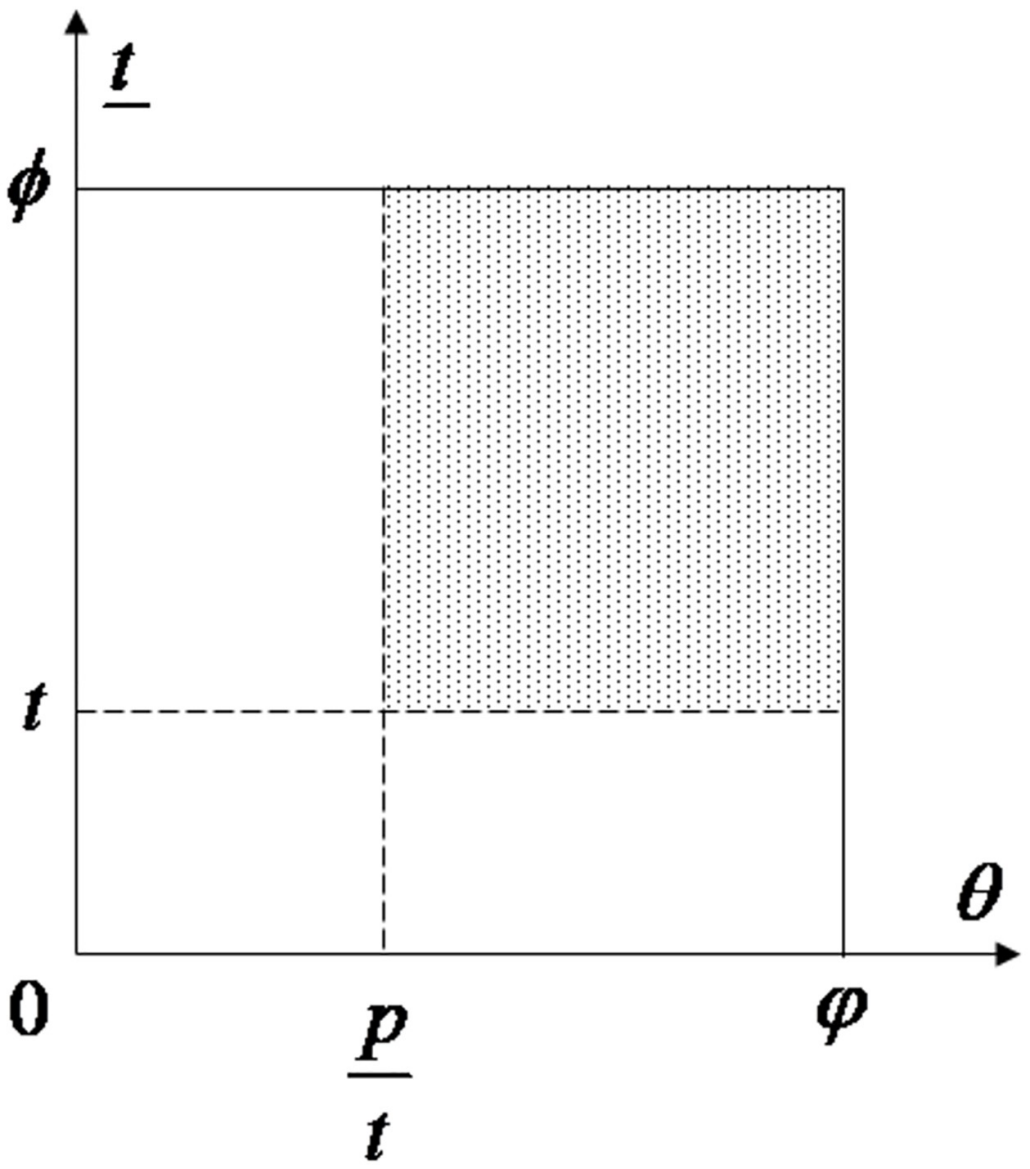
Diagram of the demand function of PEVs.

Then, the demand function of PEVs as follows (Fig 2):

$$q\left(t,p\right)=\int_{0}^{t}\frac{1}{\phi }d\underline{t}\;\int_{\frac{p}{t}}^{\varphi }\frac{1}{\varphi }d\theta =\frac{\varphi t-p}{\phi \varphi },$$ (2)

#### Enterprise profits

The profits of the PEV enterprise come from sales income and R&D subsidy income, and R&D costs and production costs are deducted.

The R&D investment function of the PEV enterprise is as follows:

$$I=kt^{2},$$ (3)

where *I* represents the R&D investment and *k* represents the R&D efficiency of the PEV enterprise. Given that $t\leq \bar{t}$ holds, where $\bar{t}\leq \phi$ means that the current ceilling of the technology level can still not reach the highest consumer technology threshold.

Furthermore, *c* is the production cost of a PEV, and *s* is the ratio of R&D result subsidy from the government.

Then, the profit function of the PEV enterprise is as follows:

$$\pi \left(t,p\right)=\left(p-c\right)\frac{\varphi t-p}{\phi \varphi }+st-I.$$ (4)

## The optimal R&D result subsidy strategies

A backward induction method was applied to solve the optimal R&D result subsidy strategies, and first, the optimal price and technology-level strategies of the PEV enterprise were solved.

Firstly, the optimal price strategies of the enterprise are as follows:

**Proposition 1** The optimal price of the PEV enterprise is $p^{*}=\frac{\varphi t+c}{2}$ with the R&D result subsidies from the government.

**Proof of Proposition 1.** Take the first derivative of Eq (4) to *p*, we can get:

$$\pi^{\prime }\left(p\right)=\frac{-2p+\varphi t+c}{\phi \varphi }.$$ (5)

Let Eq (5) = 0 and solve *p*, we get:

$$p^{*}=\frac{\varphi t+c}{2}.$$ (6)

Q.E.D.

If the highest consumer technology preference increases, all consumers have higher technology preferences as they are uniformly distributed. Consequently, the optimal PEV price depends on the consumer technology preferences, the PEV technology level, and the production cost of a PEV. From the PEV enterprise perspective, when PEV consumers have higher technology preferences, the optimal price should also be higher.

Then, solve the optimal technology-level strategies of the PEV enterprise.

From S1 File, I obtained the boundary of the R&D efficiency of the PEV enterprise, namely $k_{1}=\frac{{\left(\varphi \bar{t}-c\right)}^{2}}{4\phi \varphi {\bar{t}}^{2}}$, $k_{2}=\frac{\varphi \bar{t}-c}{4\phi \bar{t}}$, and $k_{3}=\frac{\varphi }{4\phi }$, the boundary of the R&D result subsidy, namely $s_{1}=\frac{4\phi \varphi k{\bar{t}}^{2}-{\left(\varphi \bar{t}-c\right)}^{2}}{4\phi \varphi \bar{t}}$, $s_{2}=\frac{4\phi k\left(\varphi \bar{t}+c\right)-\varphi \left(\varphi \bar{t}-c\right)}{4\phi \varphi }$, and $s_{3}=\frac{4\phi k\bar{t}-\left(\varphi \bar{t}-c\right)}{2\phi }$, and the particular value of the technology level, namely $t^{**}=-\frac{2\phi s-c}{\varphi -4\phi k}$. Further, divide the R&D efficiencies of PEV enterprises into four ranges: higher(*k* < *k*_1_), high(*k*_1_ ≤ *k* < *k*_2_), low(*k*_2_ ≤ *k* < *k*_3_), and lower(*k* ≥ *k*_1_). Based on these known analytical expressions above, we can get Proposition 2 as follows:

**Proposition 2** The optimal technology-level strategies of the PEV enterprise are as follows (Table 3):

a) When the enterprise has the higher R&D efficiency, its optimal technology-level strategy is the ceiling of the technology level, that is, $t^{*}=\bar{t}$;
b) When the enterprise has the high R&D efficiency, its optimal technology-level strategies are t* = 0 if *s* < *s*_1_ or $t^{*}=\bar{t}$ if *s* ≥ *s*_1_;
c) When the enterprise has the low R&D efficiency, its optimal technology-level strategies are t* = 0 if *s* < *kc*/*φ*, t* = *c*/*φ* if *kc*/*φ* ≤ *s* < *s*_2_, or $t^{*}=\bar{t}$ if *s* ≥ *s*_2_;
d) When the enterprise has the lower R&D efficiency, its optimal technology-level strategies are t* = 0 if *s* < *kc*/*φ*, t* = *c*/*φ* if *kc*/*φ* ≤ *s* < 2*kc*/*φ*, t* = t** if 2*kc*/*φ* ≤ *s* < *s*_3_, or $t^{*}=\bar{t}$ if *s* ≥ *s*_3_.

**Table 3:** The optimal technology-level strategies under different R&D efficiency and subsidy conditions.

Range of R&D efficiency	Classification	Scope of Subsidy	Optimal technology level
*k* < *k*_1_	higher	∀*s*	$t^{*}=\bar{t}$
*k*_1_ ≤ *k* < *k*_2_	high	*s* < *s*_1_	t* = 0
		*s* ≥ *s*_1_	$t^{*}=\bar{t}$
*k*_2_ ≤ *k* < *k*_3_	low	*s* < *kc*/*φ*	t* = 0
		*kc*/*φ* ≤ *s* < *s*_2_	t* = *c*/*φ*
		*s* ≥ *s*_2_	$t^{*}=\bar{t}$
*k* ≥ *k*_3_	lower	*s* < *kc*/*φ*	t* = 0
		*kc*/*φ* ≤ *s* < 2*kc*/*φ*	t* = *c*/*φ*
		2*kc*/*φ* ≤ *s* < *s*_3_	t* = t**
		*s* ≥ *s*_3_	$t^{*}=\bar{t}$

**Proof of Proposition 2.** See S1 File.

Q.E.D.

When the R&D efficiency of a PEV enterprise belongs to the higher range, it should always choose the highest technology level, that is, the ceiling of the technology level constrained by the current science and technology level, regardless of government subsidies. For the R&D efficiency in the other ranges, the optimal technology level depends on the subsidy ratio. If the subsidy ratio is high enough, the enterprise should also choose the ceiling; otherwise, it should select a lower or not R&D.

At last, solve the optimal technology-level strategies of the PEV enterprise.

As said in the problem description, the government wants the technology level of PEVs as high as possible, that is, to reach the ceiling of the technology level, and at the same time, the government also wants the financial investment as little as possible. Based on Proposition 1 and 2, Proposition 3 as follows:

**Proposition 3** The optimal R&D result subsidy strategies of the government are as follows (Table 4):

a) When the enterprise has the higher R&D efficiency, the government’s optimal strategy is not to provide subsidies;
b) When the enterprise has the high R&D efficiency, the optimal strategy is s* = *s*_1_;
c) When the enterprise has the low R&D efficiency, the optimal strategy is s* = *s*_2_;
d) When the enterprise has the lower R&D efficiency, the optimal strategy is s* = *s*_3_.

**Table 4:** The optimal subsidy strategies of the government under different R&D efficiencies of the PEV enterprise.

Range of R&D efficiency	Classification	The optimal R&D result subsidy ratio
*k* < *k*_1_	higher	s* = 0
*k*_1_ ≤ *k* < *k*_2_	high	s* = *s*_1_
*k*_2_ ≤ *k* < *k*_3_	low	s* = *s*_2_
*k* ≥ *k*_3_	lower	s* = *s*_3_

**Proof of Proposition 3.** According to Proposition 2, we can get the optimal R&D result subsidy strategies in Table 4.

Q.E.D.

The government should choose an optimal subsidy strategy according to the particular R&D efficiency of a PEV enterprise. The optimal subsidy strategy is not to provide subsidies for R&D efficiency in the higher range because the enterprise always chooses the ceiling of the technology level. For R&D efficiencies in the other ranges, the government can always get the enterprise to carry out R&D or raise the technology level by increasing the subsidy ratio. As long as the subsidy proportion is high enough, the enterprise will choose the ceiling of the technology level. Of course, unquestioningly increasing the subsidy proportion will cause a waste of financial funds, and the optimal ratio depends on the R&D efficiency, the ceiling of the technology level, the production cost of a PEV, and the technology threshold and preference distribution of PEV consumers.

## The impact of the consumer technology thresholds and preferences on optimal subsidy strategies

In sequence, discuss the impact of consumer technology thresholds and preferences on the optimal subsidy ratio.

Firstly, solve the impact of consumer technology thresholds on the optimal subsidy ratio. Similar to Proposition 2, Proposition 4 and 5 also classify the R&D efficiency of the PEV enterprise into four ranges: higher(*k* < *k*_1_), high(*k*_1_ ≤ *k* < *k*_2_), low(*k*_2_ ≤ *k* < *k*_3_), and lower(*k* ≥ *k*_3_). Moreover, Proposition 4 is as follows:

**Proposition 4** If PEV consumer technology thresholds rise, the government’s optimal subsidy strategies are as follows:

a) The government needs to start subsidizing PEV enterprises whose R&D efficiency is reclassified to lower ranges from the original higher range;
b) The government must increase the subsidy ratio for R&D efficiency in the original high, low, and lower ranges.

**Proof of Proposition 4**. Substitute Eq (6) into Eq (2), we get:

$$q^{*}\left(t\right)=\frac{\varphi t-c}{2\phi \varphi }.$$ (7)

According to $t^{*}=\bar{t}$ under the optimal subsidies, substitute $t^{*}=\bar{t}$ into Eqs (6) and (7), and take the first derivative of Eqs (6) and (7) to *ϕ*, we get:

$$\frac{\partial p^{*}\left(\bar{t}\right)}{\partial \phi }=0.$$ (8)

$$\frac{\partial q^{*}\left(\bar{t}\right)}{\partial \phi }<0.$$ (9)

Take the first derivative of *k*_1_, *k*_2_, *k*_3_, *s*_1_, *s*_2_, and *s*_3_ to *ϕ*, we get:

$$\frac{\partial k_{1},k_{2},k_{3}}{\partial \phi }<0.$$ (10)

$$\frac{\partial s_{1},s_{2},s_{3}}{\partial \phi }>0.$$ (11)

Q.E.D.

If the highest consumer technology threshold increases, all consumers have higher technology thresholds as they are uniformly distributed. In this case, although the optimal price of PEV enterprises is maintained, fewer consumers can accept a PEV, the sales volume of enterprises will reduce, and the profits decrease. As a result, some lower R&D efficiencies in the original higher range are reclassified to lower ranges after the increases, and these enterprises will not be able to make profits through R&D and will need subsidies from the government; for R&D efficiency in the other ranges, the old subsidy level will lead to the decline of corporate profits and the optimal technology level, and even cause enterprises to choose not to R&D. Then, the government needs to provide a higher subsidy ratio than the old to encourage them to select the ceiling of the technology level.

In contrast, if all consumers have lower technology thresholds, all PEV enterprises will hope to choose a higher technology level and profit more. As the consumer technology thresholds continue to decrease, the optimal technology level of some enterprises will reach the ceiling of the technology level, and there is no need for the government to subsidize them anymore. For the other enterprises with technology levels below the ceiling, although the government still needs to subsidize them to choose the ceiling of the technology level, the subsidy ratio can be lower than the original optimal subsidy ratio. To save on fiscal expenditures, the government should reduce the subsidy ratio.

Then, solve the impact of consumer technology preferences on the optimal subsidy ratio, and Proposition 5 is as follows:

**Proposition 5** If PEV consumer technology preferences rise, the government’s optimal subsidy strategies are as follows:

a)The government needs to stop subsidizing PEV enterprises whose R&D efficiency is reclassified to the higher range from lower original ranges;
b) The government must reduce the subsidy ratio for R&D efficiency in the original high, low, or lower ranges.

**Proof of Proposition 5.** According to $t^{*}=\bar{t}$ under the optimal subsidies, substitute $t^{*}=\bar{t}$ into Eqs (6) and (7), and take the first derivatives of Eqs (6) and (7) to *φ*, we get:

$$\frac{\partial p^{*}\left(\bar{t}\right)}{\partial \varphi }>0,$$ (12)

$$\frac{\partial q^{*}\left(\bar{t}\right)}{\partial \varphi }>0.$$ (13)

Take the first derivatives of *k*_1_, *k*_2_, *k*_3_, *s*_1_, *s*_2_, and *s*_3_ to *φ*, we get:

$$\frac{\partial k_{1},k_{2},k_{3}}{\partial \varphi }>0,$$ (14)

$$\frac{\partial s_{1},s_{2},s_{3}}{\partial \varphi }<0.$$ (15)

Q.E.D.

If each PEV consumer has a higher technology preference, the optimal PEV price and the sales volume of the PEV enterprise will increase, leading to more profits. Similar to the situation where the consumer technology thresholds reduce, as the consumer technology preferences continue to increase, the optimal technology level of some enterprises will reach the ceiling of technology level, and there is no need for the government to subsidize them anymore. For the other enterprises with technology levels below the ceiling, although the government still needs to subsidize them to choose the ceiling of the technology level, the subsidy ratio can be lower than the original optimal subsidy ratio. Moreover, the government should reduce the subsidy ratio to save fiscal expenditures.

## Numerical simulation

Firstly, the numerical simulation compared the effect of the model considering the anxiety coefficient with the model considering the consumer technology thresholds, that is, the model in this paper, under the same subsidy ratio. Then, it visualized the influence of changes in consumer technology thresholds and preferences on the optimal subsidy ratio. Both were implemented by Mathematica 11.

For comparing the effect of the model considering the anxiety coefficient, that is, Model 1, with the model considering the consumer technology thresholds, that is, Model 2, under the same subsidy ratio, replace the consumer technology thresholds and preferences in Model 2 with an anxiety coefficient and constructed Model 1 (S2 File), as the significant difference between the model in the reference and Model 2, which makes it difficult to compare [3, 12, 24].

**Fig 3:**
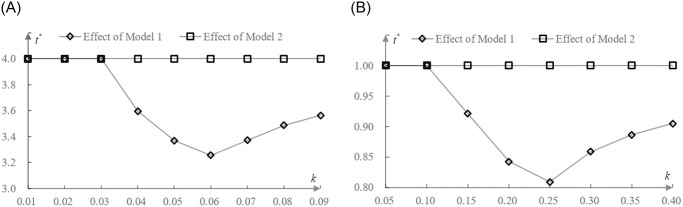
**A.** The comparison of technology level strategies of the PEV enterprise between Model 1 and 2 with higher technology thresholds. **B.** The comparison of technology level strategies of the PEV enterprise between Model 1 and 2 with lower technology thresholds.

Let *ϕ* = 4, *φ* = 1, *c* = 2, $\bar{t}=4$, and *k* = 0.01, 0.02, 0.03, 0.04, 0.05, 0.06, 0.07, 0.08 *and* 0.09 respectively, and get the optimal subsidy ratio for Model 2 according to Proposition 3 (S2 File). Under the same parameter conditions, make the subsidy ratio *s* of Model 1 equal to the optimal subsidy ratio of Model 2 and solve the optimal technology level strategies of the PEV enterprise in Model 1 (S2 File). The comparison of technology-level strategies of the PEV enterprise between Model 1 and 2 under the same subsidy ratio is shown in Fig 3A.

Similarly, let *ϕ* = 1, *φ* = 1, *c* = 0.5, $\bar{t}=1$, and *k* = 0.05, 0.10, 0.15, 0.20, 0.25, 0.30, 0.35, *and* 0.40 respectively, solve and compare the technology level strategies of Model 1 and 2 under the same subsidy ratio in Fig 3B (S2 File).

In Fig 3A, the technology threshold parameter *ϕ* = 4 is higher than that in Fig 3B, that is, *ϕ* = 1. When the enterprise has the R&D efficiency *k* = 0.01, 0.02 *or* 0.03, the optimal subsidy ratio for Model 2, that is, *s* = 0.0000, 0.0175 *or* 0.0575, makes the enterprise select the ceiling of technology level in Model 1. When the R&D efficiency of enterprises continues to decrease, the optimal subsidy ratio of Model 2 cannot allow the enterprises in Model 1 to choose the ceiling of the technology level.

In Fig 3B, the technology threshold parameter is lower, and the unit production cost and the ceiling of the technology level are also lower, that is, *ϕ* = 1, *c* = 0.5 and $\bar{t}=1$. Similar to the results shown in Fig 3A, the optimal subsidy ratio of Model 2 may not necessarily achieve the best incentive effect of Model 1. When the enterprise has the R&D efficiency *k* = 0.05 *or* 0.10, the optimal subsidy ratio for Model 2, that is, *s* = 0.0000 *or* 0.0375, makes the enterprise select the ceiling of technology level in Model 1. When the R&D efficiency of enterprises continues to decrease, the optimal subsidy ratio of Model 2 cannot allow the enterprises in Model 1 to choose the ceiling of technology level.

Both Fig 3A and 3B show that there is usually a significant difference in the optimal subsidy ratio between Model 1 and Model 2, indicating that a fundamental difference exists in modeling anxiety issues from the perspectives of consumer technology threshold or anxiety coefficient. Therefore, it is necessary to construct the demand part of the game model based on the consumer technology threshold, considering the impact of anxiety issues on the government’s optimal subsidy strategies.

**Fig 4:**
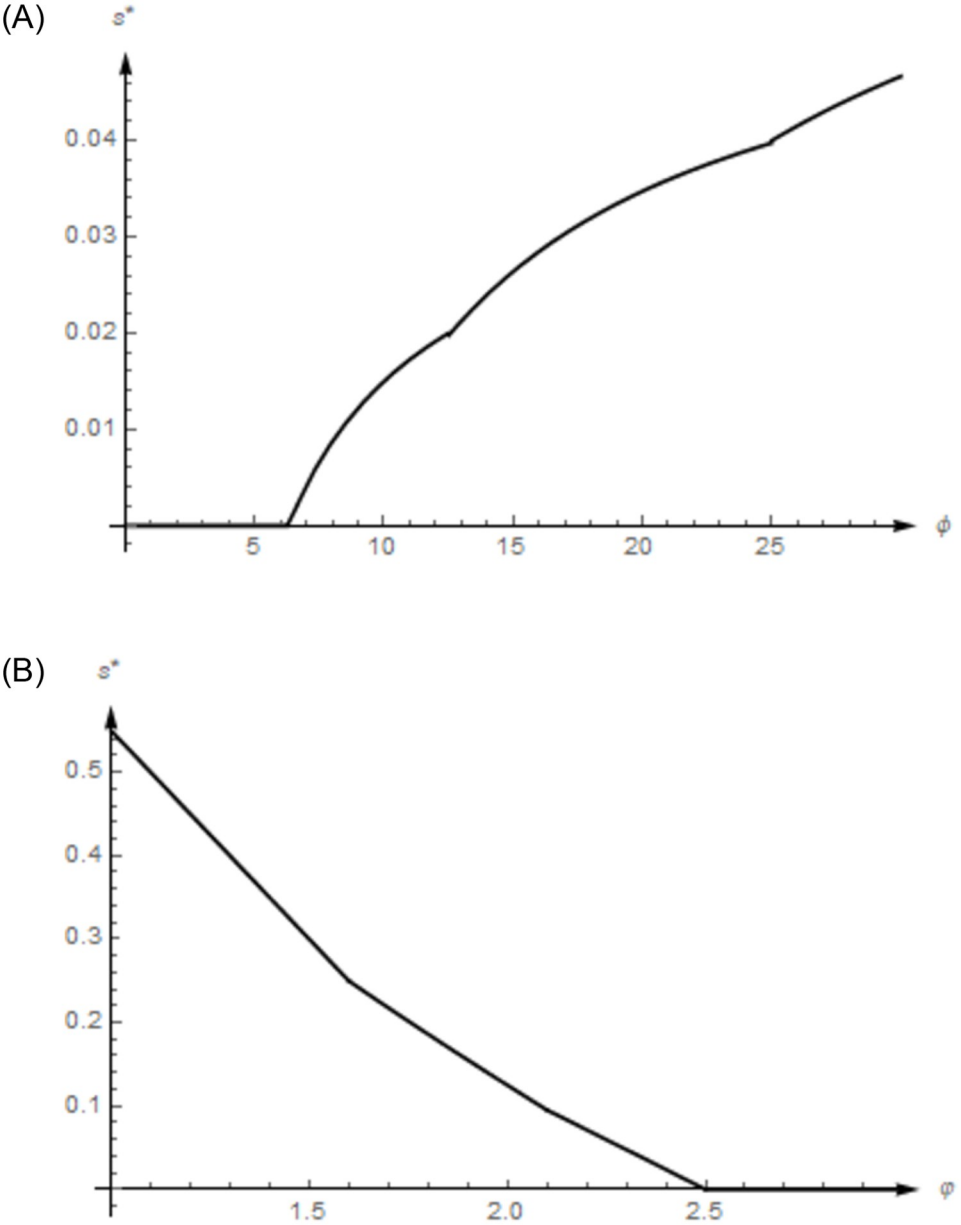
**A.** The influence of consumer technology thresholds on the subsidy ratio. **B.** The influence of consumer technology preferences on the subsidy ratio.

Then, draw the influence of changes in consumer technology thresholds and preferences on the optimal subsidy ratio. According to the optimal subsidy ratios in Proposition 3, namely *s*_1_, *s*_2_ and *s*_3_, let *k* = 0.01, *φ* = 1, *c* = 2, and $\bar{t}=4$ and see the influence of consumer technology thresholds on optimal subsidy ratios as the highest consumer technology threshold *ϕ* increases from *ϕ* = 4 in Fig 4A.

Similarly, let *k* = 0.1, *φ* = 1, *c* = 2, and $\bar{t}=4$, and see the influence of consumer technology preferences on optimal subsidy ratios s* as *φ* increases from *φ* = 1 in Fig 4B.

In Fig 4A, the technology thresholds of PEV consumers continues to grow, that is, the highest consumer technology threshold *ϕ* increases from *ϕ* = 4 to *ϕ* = 30, the optimal subsidy ratio of the government remains unchanged when *ϕ* ∈ [4, 6.2) and it also increases when *ϕ* ∈ [6.2, 10.5), *ϕ* ∈ [10.5, 24.7) and *ϕ* ∈ [24.7, 30] accordingly. However, the growth rate of the optimal subsidy ratio varies, with the fastest growth in *ϕ* ∈ [6.2, 10.5), followed by *ϕ* ∈ [10.5, 24.7), and the slowest in *ϕ* ∈ [24.7, 30]. Overall, the limited increase in consumer technology thresholds does not necessarily lead to an increase in the optimal subsidy ratio. However, as long as the consumer technology thresholds are high enough, the government must correspondingly raise the optimal subsidy ratio to maintain the maximum incentive effect of subsidy policies, that is, to allow the enterprises to choose the ceiling of technology.

In Fig 4B, the technology preferences of PEV consumers continues to grow, that is, the highest consumer technology threshold *φ* increases from *φ* = 1 to *φ* = 3, the optimal subsidy ratio of the government decreases accordingly when *φ* ∈ [1.0, 1.6), *φ* ∈ [1.6, 2.1) and *φ* ∈ [2.1, 2.5), and then it remains unchanged when *φ* ∈ [2.5, 3.0]. However, the reduction rate of the optimal subsidy ratio varies, with the fastest reduction in *φ* ∈ [1.0, 1.6), followed by *φ* ∈ [1.6, 2.1), and the slowest in *φ* ∈ [2.1, 2.5). In general, the higher the technology preferences of PEV consumers, the lower the optimal subsidy ratio until the enterprise’s technology level reaches the ceiling and subsidies are unnecessary.

## Discussions

The solution results of the optimal technology level strategies reflect the logic of selecting the technology level for the PEV enterprise. Generally speaking, selecting the technology level depends on the technology’s positive or negative marginal profit. If improving technology can generate profits, the company will raise its technology; otherwise, it will reduce it. Technology’s marginal profit depends on comparing costs and benefits brought by improving technology. The R&D efficiency of the enterprise significantly affects the costs of technology improvement, which largely determines the size and trend of the marginal profit of the technology. For example, when the R&D efficiency of the enterprise is in the higher range, the marginal profit of the technology is positive and continues to grow. Regardless of whether government subsidies or not, the company will pursue higher technology and higher profits. Due to the limitations of current scientific and technological levels, the enterprise will choose the current ceiling of the technology.

The solution results of the optimal R&D result subsidy strategies reflect the principle that changes in subsidy ratio affect the selecting of technology level of the PEV enterprise. Government subsidies are new revenue sources for improving technology, thus changing the trend of marginal profits in technology. By adjusting the subsidy ratio, the government can make the enterprise choose the current ceiling of the technology. For example, when the R&D efficiency is in the high range, the marginal profit of the technology first is positive and then negative with the technology improvement. In order to enable the enterprise to choose the technology ceiling, the government needs to increase the subsidy ratio so that the enterprise can maintain positive technological marginal profits at a higher technology level and select the higher technology level. In other words, a sufficiently high subsidy ratio can enable the company to choose the current technological ceiling. Moreover, once the company chooses the technology ceiling, if the government continues to increase the subsidy ratio, that is, the optimal subsidy ratio, it will waste fiscal funds.

Consumer technology thresholds affect the optimal subsidy ratio by influencing the marginal benefits of technology improvement. Increasing consumer technology thresholds will lead to lower sales and profits for the PEV enterprise at the same optimal price, that is, lower or even negative technological marginal profits. Therefore, increasing consumer technology thresholds often leads the company to choose lower technology levels. In order to achieve the desired incentive effect, the government will need to increase the optimal subsidy ratio correspondingly to raise the marginal benefits of the technology. Moreover, if the R&D efficiency of the enterprise is in the higher range, the limited increase in consumer technology thresholds has a slight negative impact on the marginal profit of technology. The enterprise still tends to choose the highest possible level of technology, that is, the current technology ceiling, and the government still does not need to provide subsidies.

Consumer technology preferences also affect the optimal subsidy ratio by influencing the marginal benefits of technology improvement. The rise in technology preferences will lead to higher sales and profits for PEV enterprises at a higher optimal price, which means the enterprise will have higher technological marginal profits. Therefore, the rise in consumer technology preferences often leads companies to choose a higher technology level, and the government needs to lower the optimal subsidy ratio correspondingly to save on subsidy expenses.

The findings of this paper can be applied to guide governments around the world that have already and are preparing to adopt an R&D result subsidy policy to promote PEV technology progress, whose PEV market faces typical anxiety problems. Governments of various countries can consider PEV enterprises and consumer characteristics based on the theoretical model presented in this article to obtain the optimal subsidy ratio. For example, the governments of the United States, the United Kingdom, or 5 Nordic countries can refer to a general travel range of their PEV consumers to distinguish their respective consumer technology threshold characteristics [38, 39]. Of course, it may be challenging to quantify all enterprise and consumer characteristics reasonably in practical applications. For example, PEV consumers have multiple dimensions of technological thresholds and significant differences in the distribution of technological thresholds. Therefore, the value discovered in this study will be reflected more in its significance in theoretical guidance.

This article also has some limitations. Considering the potential challenges in solving the model, the model did not consider the competition among PEV enterprises in reality, nor did it consider the more complex distribution of consumer technology thresholds, which may be a more realistic situation. Due to the limitations of the established model and analysis, this article did not discuss the potential unintended consequences of the R&D result subsidy policy, such as market distortions or the risk of dependency on government support by PEV enterprises. In the future, improving the models and expanding the breadth and depth of the research will be carried out to address these limitations.

## Conclusions

This paper analyzed the anxiety issues from the perspective of PEV consumer technology thresholds, constructed a sequential game model among the government, the PEV enterprise, and consumers with the heterogeneous technology thresholds and preferences, and obtained the optimal R&D result subsidy strategies and the impact of technology thresholds and preferences on them, which has great significance for analyzing anxiety issues and optimizing government R&D result subsidy policies.

The government should subsidize PEV enterprises other than those with R&D efficiency in the higher range. They pursue higher technology and corresponding higher profits, so they always choose the technology ceiling in the current stage, and the government does not need to waste fiscal funds to incentivize them. For the other enterprises, the lower profits have led to a lack of motivation to pursue the higher technology, and the R&D result subsidy policy would incentivize them to choose higher technology by increasing their profits on the higher technology level. Furthermore, the optimal subsidy ratio must consider consumer technology threshold and preference conditions to ensure the desired incentive effect with minimal fiscal expenditure.

From the perspective of PEV consumer technology thresholds, a more severe anxiety problem means higher consumer technology thresholds in the PEV market, which usually has a more significant negative impact on the R&D result subsidy policy. The increase in consumer technology thresholds directly leads to lower sales and profits for PEV enterprises at the same optimal price. In other words, it leads to decreased or even negative marginal profits for enterprises to improve their technology and companies choosing lower technology levels. In contrast, if the marginal profit of improving technology for the PEV enterprise is relatively high, the limited increase in consumer technology thresholds will not affect the enterprise’s choice of the ceiling of technology level at the current stage. The government still does not need subsidies for PEV enterprises.

The government should adjust the optimal subsidy ratio based on the specific impact of anxiety problems on technology marginal profits and the enterprise’s technology choice. When severe anxiety problems or low technology marginal profits, the subsidy ratio should be increased on time to ensure incentive effectiveness. The government should reduce the subsidy ratio to raise incentive efficiency when mild anxiety problems or high technology marginal profits.

On the contrary, the higher the technology preferences of PEV consumers, the lower the optimal subsidy ratio is usually. Increasing consumer technology preferences will lead to higher sales and profits for PEV enterprises at a higher optimal price. It will also lead to higher marginal profits for the enterprises improving their technology and the companies choosing higher technology levels. However, if the enterprise has selected the ceiling of technology level at the current stage, government subsidies cannot enable it to improve its technology further.

On the one hand, the government can promptly adjust the optimal subsidy ratio to ensure the effectiveness and efficiency of incentives for higher technology preferences. On the other hand, the government can also improve consumer technology preferences through various means, such as cultivating consumer recognition of green technologies, to stimulate PEV enterprise’s R&D and sales.

## Supporting information

S1 File Appendix A. (DOCX)

S2 File Appendix B. (DOCX)
