# Supplementary material for: Study on R&D result subsidy strategies for PEV enterprises based on heterogeneous consumer technology thresholds and preferences under anxiety issues
Source: PLoS One. 2025 Feb 18;20(2):e0314476. doi: 10.1371/journal.pone.0314476 (PMC11835243; doi:10.1371/journal.pone.0314476)
Supplement: S1 File — (DOCX) [file pone.0314476.s001.docx]

**Appendix A**

Substitute Eq(6) into Eq(4), and transform the profit function of the PEV enterprise into the form as below:

.

From Eq(2), we get:

.

According to Eq(A.1) and Eq(A.2), we know that $\pi\left(t\right)$ is continuous and differentiable only in and , and the profit function $\pi\left(t\right)$ should be corrected as:

,

and take the first and second derivatives of Eq(A.3) to $t$ when , we get:

,

.

From Eq(A.3)-Eq(A.5), we get:

,

,

$\left\{\begin{matrix} \pi''\left(t\right)<0\text{ }k>\frac{\varphi}{4\phi} \\ \pi''\left(t\right)\geq0\text{ }k<\frac{\varphi}{4\phi} \end{matrix}\right.$,

from (A.6)-(A.8), we further get:

,

,

and then discuss the optimal strategies of technology level in 6 cases:

①

Denote $G_{1}\left(s\right)=\pi\left(\bar{t}\right)$, and we get:

.

It can be proved that $G_{1}\left(s\right)$ is a monotone-increasing function. Let $G_{1}\left(s\right)=0$ and solve $s$, we get the unique root as:

.

From $k<\frac{\varphi}{4\phi}$ and Eq(A.12), we get:

,

,

from Eq(A.12)-Eq(A.14), we get the optimal technology level as:

.

②

Denote , and we get:

.

It can be proved that $G_{2}\left(s\right)$ is a monotone-increasing function. Let $G_{2}\left(s\right)=0$ and solve $s$, we get the unique root as:

.

From $k<\frac{\varphi}{4\phi}$ and Eq(A.17), we get:

,

,

from Eq(A.17)-Eq(A.19), we get the optimal technology level as:

.

③

From Eq(A.9), we get the optimal technology level as:

$t^{*}=\bar{t}$.

④

From Eq(A.10), we get the optimal technology level as:

$t^{*}=0$.

⑤

From Eq(A.10), we get the optimal technology level as:

.

⑥

Let $\pi'\left(t\right)=0$ and solve $t$, we get:

,

and denote $G_{3}\left(s\right)=\bar{t}-t^{**}$, we get:

.

Similarly, $G_{3}\left(s\right)$ is a monotone-increasing function. Let $G_{3}\left(s\right)=0$ and solve $s$, we get the unique root as:

.

From $k>\frac{\varphi}{4\phi}$ and Eq(A.26), we get:

,

from Eq(A.26)-Eq(A.27), we get the optimal technology level as:

.

Denote , $k_{2}=\frac{\varphi\bar{t}-c}{4\phi\bar{t}}$, and $k_{3}=\frac{\varphi}{4\phi}$, and synthesize the optimal technology level from ①-⑥, we get the optimal strategies of the technology level as Table 3.
