# Supplementary material for: Study on R&D result subsidy strategies for PEV enterprises based on heterogeneous consumer technology thresholds and preferences under anxiety issues
Source: PLoS One. 2025 Feb 18;20(2):e0314476. doi: 10.1371/journal.pone.0314476 (PMC11835243; doi:10.1371/journal.pone.0314476)
Supplement: S2 File — (DOCX) [file pone.0314476.s002.docx]

**Appendix B**

For building a demand model of a PEV consumer basing on an anxiety coefficient, define $\lambda$ as the anxiety coefficient instead of the consumer technology thresholds $\underline{t}$ and preferences $\theta$, which distributed uniformly from 0 to 1.

Then, the consumer purchase decision function $Eq\left(1\right)$ changes as follows:

$U\left(t\right)=\lambda t-p$.

The demand function $Eq\left(2\right)$ changes as follows:

$q\left(t,p\right)=\int_{\frac{p}{t}}^{1} d\lambda=\frac{t-p}{t}$.

The profit function of the PEV enterprise changes as follows:

$\pi\left(t,p\right)=\left(p-c\right)\frac{t-p}{t}+st-I$.

Take the first derivative of $Eq\left(B.3\right)$ to $p$, we can get:

$\pi'\left(p\right)=\frac{t-2p+c}{t}$.

Let $Eq\left(B.4\right)=0$ and solve $p$, we get:

$p^{*}=\frac{t+c}{2}$.

From $Eq\left(B.2\right)$ and $Eq\left(B.5\right)$, we get:

$q^{*}\left(t\right)<0,\text{ }if\text{ }t<c$.

Substitute $Eq\left(B.5\right)$ into $Eq\left(B.3\right)$, and transform the profit function of the PEV enterprise into the form as below:

$\pi\left(t,p\right)=\frac{\left(t-c\right)^{2}}{4t}+st-kt^{2}$.

According to $Eq\left(B.6\right)$ and $Eq\left(B.7\right)$, we know that $\pi\left(t\right)$ is continuous and differentiable only in $\left[0,c\right)$ and $\left(c,\bar{t}\right]$, and the profit function $\pi\left(t\right)$ should be corrected as:

$\pi\left(t\right)=\left\{\begin{matrix} -kt^{2}\text{ }t<c \\ -kt^{2}+\frac{1+4s}{4}t+\frac{c^{2}}{4t}-\frac{c}{2}\text{ }c<t\leq\bar{t} \end{matrix}\right.$,

and take the first, second and third derivatives of $Eq\left(B.8\right)$ to $t$ when $c<t\leq\bar{t}$, we can get:

$\pi'\left(t\right)=-2kt+\frac{1+4s}{4}-\frac{c^{2}}{4t^{2}}$,

$\pi''\left(t\right)=-2k+\frac{c^{2}}{2t^{3}}$.

Let , , $c=2$, $\bar{t}=4$, and $k=0.01,\text{ }0.02,\text{ }0.03,\text{ }0.04,\text{ }0.05,\text{ 0.06,}$ $\text{0.07, 0.08 }and\text{ }0.09$respectively, and solve the optimal subsidy ratio of Model 2 according to Proposition 3 (Table B1).

**Table B1. The comparison of technology level strategies of the PEV enterprise between Model 1 and 2 with higher technology thresholds**

| R&D efficiency | Subsidy ratio | Effect of Model 1 | Effect of Model 2 |
| --- | --- | --- | --- |
| $k=0.01$ | $s=0.0000$ | $t^{*}=4.0000$ | $t^{*}=4$ |
| $k=0.02$ | $s=0.0175$ | $t^{*}=4.0000$ | $t^{*}=4$ |
| $k=0.03$ | $s=0.0575$ | $t^{*}=4.0000$ | $t^{*}=4$ |
| $k=0.04$ | $s=0.1150$ | $t^{*}=3.5957$ | $t^{*}=4$ |
| $k=0.05$ | $s=0.1750$ | $t^{*}=3.3690$ | $t^{*}=4$ |
| $k=0.06$ | $s=0.2350$ | $t^{*}=3.2553$ | $t^{*}=4$ |
| $k=0.07$ | $s=0.3100$ | $t^{*}=3.3717$ | $t^{*}=4$ |
| $k=0.08$ | $s=0.3900$ | $t^{*}=3.4856$ | $t^{*}=4$ |
| $k=0.09$ | $s=0.4700$ | $t^{*}=3.5622$ | $t^{*}=4$ |

Then, according to $Eq\left(B.8\right)-\left(B.10\right)$, solve the optimal technology level strategies of the PEV enterprise of Model 1 under the same parameter values with Model 2, and compare technology level strategies of the PEV enterprise as follows:

Similarly, let , , $c=0.5$, $\bar{t}=1$, and $k=0.10,\text{ }0.15,\text{ }0.20,\text{ }0.25,\text{ 0.30, 0.35 }and\text{ }0.40$ respectively, and compare technology level strategies of the PEV enterprise as follows (Table B2):

**Table B2. The comparison of technology level strategies of the PEV enterprise between Model 1 and 2 with lower technology thresholds**

| R&D efficiency | Subsidy ratio | Effect of Model 1 | Effect of Model 2 |
| --- | --- | --- | --- |
| $k=0.05$ | $s=0.0000$ | $t^{*}=1$ | $t^{*}=1$ |
| $k=0.10$ | $s=0.0375$ | $t^{*}=1$ | $t^{*}=1$ |
| $k=0.15$ | $s=0.1000$ | $t^{*}=0.9211$ | $t^{*}=1$ |
| $k=0.20$ | $s=0.1750$ | $t^{*}=0.8422$ | $t^{*}=1$ |
| $k=0.25$ | $s=0.2500$ | $t^{*}=0.8090$ | $t^{*}=1$ |
| $k=0.30$ | $s=0.3500$ | $t^{*}=0.8587$ | $t^{*}=1$ |
| $k=0.35$ | $s=0.4500$ | $t^{*}=0.8863$ | $t^{*}=1$ |
| $k=0.40$ | $s=0.5500$ | $t^{*}=0.9045$ | $t^{*}=1$ |
